# Polyethylene glycol as an improved barrier to prevent fleeing in *C. elegans*

**DOI:** 10.17912/micropub.biology.001288

**Published:** 2024-08-08

**Authors:** Safa Beydoun, Aditya Sridhar, Mira Bhandari, Elizabeth S Kitto, Scott F Leiser

**Affiliations:** 1 Molecular and Integrative Physiology, University of Michigan–Ann Arbor, Ann Arbor, Michigan, United States; 2 Molecular, Cellular and Developmental Biology, University of Michigan–Ann Arbor, Ann Arbor, Michigan, United States; 3 Department of Internal Medicine, University of Michigan–Ann Arbor, Ann Arbor, Michigan, United States

## Abstract

*Caenorhabditis elegans* studies can be constrained by worms escaping standard solid nematode growth medium (NGM) plates. When worms are in search of food or are avoiding pathogens, chemicals, and environmental stressors, they often exhibit a behavior known as “fleeing”. Palmitic acid (PA) is sometimes used as a barrier “fence” to reduce fleeing under limited food and oxygen conditions. Here, we evaluate the efficacy of palmitic acid, polyethylene glycol (PEG) and copper as potential barriers to reduce fleeing under various environmental conditions. Our results indicate that PA and PEG each reduce fasted flee rate and do not obviously alter overall health and lifespan of the worms, while copper blunts worm growth and development. We also find that PEG is a more optimal tool than PA since it is more effective in fasted conditions, reduces flee rate in a pathogenic environment, and does not alter worm size.

**Figure 1. Use of barriers to prevent fleeing in C. elegans:**
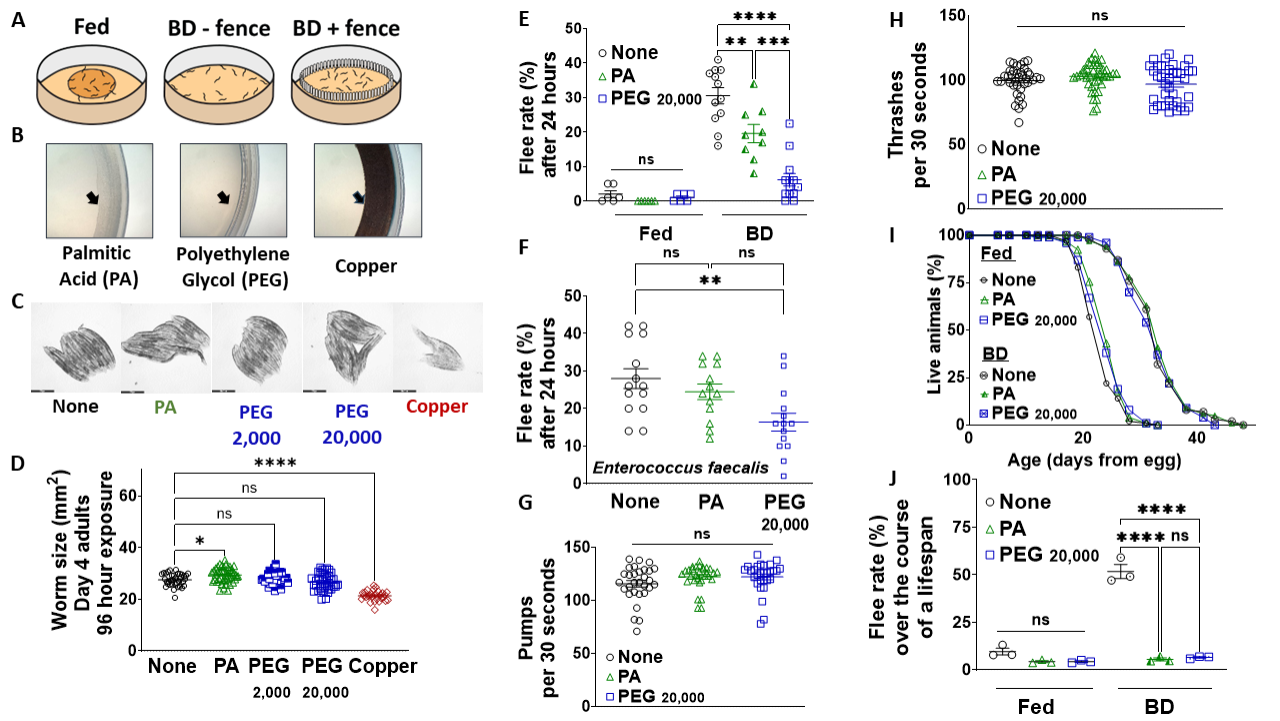
( **A** ) Schematic of worms on plates with food (left), no food (middle), and no food with a barrier/fence (right). ( **B** ) Representative images of palmitic acid (PA), polyethylene glycol (PEG), and copper fences (edge of barrier is shown by an arrow) placed on the edge of NGM plates. Note: PEG is not visible when it dries. ( **C** ) Representative bright-field images of wildtype fed worms following a 96-hour exposure to PA, PEG 2,000, PEG 20,000, and copper as compared to a no fence control. ( **D** ) Quantification of images in (D) to determine the effect of each fencing tool on worm size. ( **E** ) Flee rate of day 1 adult wildtype worms under 24 hours of fed and fasted conditions with PA and PEG 20,000 fences as compared to a no fence control. ( **F** ) Flee rate of day 1 adult wildtype worms after a 24-hour exposure to pathogenic bacteria *Enterococcus faecalis* with PA and PEG 20,000 fences as compared to a no fence control. ( **G** ) Pharyngeal pumping and ( **H** ) thrashing rates of wildtype worms after a 24-hour exposure to PA and PEG 20,000 as compared to no fence fed control. ( **I** ) Lifespan of wildtype fed and DR (bacterial deprivation, or BD) worms at 20°C with PA and PEG 20,000 fences as compared to a no fence control. ( **J** ) Quantification of flee rate during lifespans shown in (I). One-way ANOVA with Tukey Post-Hoc analysis was used to derive p-values for worm size, flee rate, pumping and thrashing comparisons. Log-rank test was used to derive p-value for lifespan comparisons. All error bars shown in the figures represent the standard error of the mean (SEM). ns, not significant, * denotes p-value < 0.05, ** denotes p-value < 0.01, *** denotes p-value <0.001 and **** denotes p-value < 0.0001.

## Description

The nematode *Caenorhabditis elegans* is a valuable model organism in stress response, pathogen, and aging studies due to its short lifespan, genetic tractability, and ease of maintenance (Tissenbaum, 2015) . Several conserved longevity pathways were initially discovered in *C. elegans* (Garigan et al., 2002, Houthoofd et al., 2005, Olsen et al., 2006a, Olsen et al., 2006b). Standard growth and maintenance of *C. elegans* utilizes nematode growth medium (NGM) in a solid agar mixture on a Petri dish, typically 60 mm in diameter. When worms on these plates are faced with adverse conditions such as dietary restriction (DR), they begin a multi-stage search for food known as foraging. This behavior is characterized by an initial intensive “local” search, followed by a larger “global” search (Calhoun et al., 2014) . In a DR environment, foraging is inherently unable to yield a local food source. Once this local search is exhausted, individual worms will often “flee” or crawl off the agar to conduct a global search. Such a search can yield one of three outcomes: (1) a return to the NGM agar, (2) burrowing to the interior of the agar, or (3) desiccation on the side of the dish. While the first outcome is largely benign, both burrowing and desiccation lead to a reduction in the experimental population. The ratio of worms that flee the Petri dish relative to the initial population is known as the flee rate. Prior studies have reported this percentage as low as 50% (Kaeberlein et al., 2006) and as high as 70% (Sutphin and Kaeberlein, 2009) over the course of a lifespan under DR. Not only do worms roam the environment in search of food, but they also flee from pathogens (e.g., *Pseudomonas aeruginosa* (Kaletsky et al., 2020) and *Enterococcus faecalis* (Yuen and Ausubel, 2018) ), chemicals (e.g., osmotic (Culotti and Russell, 1978) and oxidative (Oh et al., 2015) ), and environmental stressors (e.g., thermal (Mohammadi et al., 2013) and hypoxic (Zhao et al., 2022) ).

A palmitic acid (PA) barrier has been used as an aversive barrier or “fence” ( **Fig. 1A** ) to reduce fleeing under dietary restriction and in a low oxygen (hypoxic) environment (Fawcett et al., 2012) . In this study, we compare the efficacy of PA with two novel barrier “fences” consisting of polyethylene glycol (PEG) and solid copper ( **Fig. 1A -B** ). PEG has been used as a tool to immobilize worms for live imaging (Burnett et al., 2018, Pittman et al., 2017) , while copper has been used in CuSO _4_ solution in multi-well plates to prevent worms from escaping their wells (Churgin et al., 2017) . PA was dissolved in 100% ethanol while PEG was dissolved in M9 buffer (Stiernagle, 2006) . All fencing techniques were prepared 24 hours before the onset of experiments. To initially test whether they affect worm physiology, we grew wild-type worms with these fences from egg for 96 hours. On normal NGM growth plates, we found that copper blunted worm growth as shown by decreased worm size as compared to the no fence control, while PA led to a small but statistically significant increase in worm size ( **Fig. 1C -D** ). PEG did not alter *C. elegans* size compared to the control unfenced condition. This was consistent across the two available sizes of PEG, 2,000 and 20,000 ( **Fig. 1C -D** ). Since copper slowed growth, may have leached into the agar, and has been shown to lead to toxicity (Moyson et al., 2019, Moyson et al., 2018, Song et al., 2014) , it was excluded from further evaluation. We also chose to move forward with the larger PEG molecules to best prevent the possibility that the worms consume PEG. Thus, we further tested PA and PEG 20,000 barrier fences in comparison with control conditions.

We next compared the flee rate of worms fenced with PA and PEG under various conditions. When worms were placed in a fed ( *E. coli* OP50 ) environment, we did not see a difference in flee rate among the PA, PEG and no fence control, as < 3% fleeing was observed ( **Fig. 1E** ). However, upon exposure to a DR environment, in this case 24 hours of fasting, the flee rate of day 1 adult wildtype worms increased to 30% in the absence of a fence, but this rate was lower using a PA (20%) and a PEG 20,000 (5%) fence ( **Fig. 1E** ). This result suggests that both PA and PEG limit flee rate under DR with PEG being more effective. Since worms also flee when exposed to a repellant/pathogen, we next tested the efficacy of PA and PEG in preventing fleeing when worms are exposed to the pathogen, *Enterococcus faecalis* (EF). In the absence of a fence, the flee rate of worms after 24 hours on EF is about 30% and is not improved with the addition of a PA fence ( **Fig. 1F** ). However, a PEG fence significantly reduces the flee rate of worms exposed to the pathogenic bacteria by ~10% ( **Fig. 1F** ). These results suggest that PEG is a more effective barrier in a pathogenic environment.

Since the goal of a barrier is to maintain worms on the plate without changing their physiology, we next tested whether the barriers affect measures of health and longevity. We placed day 1 adult worms on condition plates for 24 hours and tested the effect of PA and PEG on pumping and thrashing. Neither pumping ( **Fig. 1G** ) nor thrashing ( **Fig. 1H** ) was altered by PA and PEG exposure. We were also interested in testing whether long term exposure to PA and PEG influenced lifespan under fed and dietary restriction conditions. Similar to the health measures, long term exposure to PA and PEG did not have a significant effect on fed or DR (bacterial deprivation, or BD (Kaeberlein et al., 2006) ) lifespan ( **Fig. 1I** ), which was extended compared to control fed in all conditions. This result suggests that neither PA nor PEG modify the rate of aging in normal fed and DR environments.

Together, our results indicate that both PA and PEG are viable tools to prevent fleeing in *C. elegans ,* with PEG being more effective across conditions. PEG reduces the flee rate beyond that exhibited by PA and does so consistently across conditions of BD and EF. While PA does not modulate lifespan in control fed and DR conditions, its small effect on worm size does raise concerns that the compound may modify growth and/or metabolism and could confound studies when PA is employed as a barrier. We also find that maintaining additional worms on the plate through fencing in DR lifespans did not significantly affect the lifespan, suggesting that the worms who flee are not greatly “healthier” or “sicker” than worms who stay on the plate. 50% of worms fled throughout the lifespan when exposed to BD without a fence while less than 10% of worms fled under BD with either PEG or PA as a barrier ( **Fig. 1J** ). Together, these results suggest that PEG can be a useful tool for maintaining worms in environments where fleeing is problematic.

## Methods

**Strains and growth conditions**

Standard *Caenorhabditis elegans* cultivation procedures were used as previously described (Kaeberlein et al., 2006, Smith et al., 2008) . Briefly, N2 wild type worms were maintained on solid nematode growth media (NGM) seeded with 200 μL live *E. coli* OP50 (OD _600_ 3.0) throughout life and housed in a 20°C Percival incubator. All experiments were conducted at 20°C unless stated otherwise.

**Fencing interventions**

Palmitic acid (PA): 0.5 g palmitic acid (MP Biomedicals 100905) was dissolved in 50 mL of 100% ethanol. 80 μL of the PA solution was added to the edge of the agar and allowed to dry for 24 hours before use. Polyethylene glycol 2,000 (PEG 2,000): 5 g of PEG 2,000 (Alfa Aesar 25322-68-3) was dissolved in 25 mL of M9 buffer (Stiernagle, 2006) (3 g KH _2_ PO _4_ , 6 g Na _2_ HPO _4_ , 5 g NaCl, 1 mL 1 M MgSO _4_ , H _2_ O to 1 liter. Sterilized by autoclaving). Polyethylene glycol 20,000 (PEG 20,000): 5 g of PEG 20,000 (Thermo Scientific A17925.30) was dissolved in 25 mL of M9 buffer. 160 μL of each PEG solution was added to the edge of the agar plates and allowed to dry for 24 hours before use. PEG is more viscous than PA and requires a larger volume to fully fence the plates. Copper: Copper washers (0.5 cm in width) that fit inside a 60 mm plate were autoclaved then individually heated before adding on top of the agar to create a seal and prevent worms from crawling between the copper and the agar. Copper plates were also prepared 24 hours before use.

**Worm size**

N2 wildtype worms were synchronized by a timed-egg-lay on NGM plates. The animals (n=50) were allowed to develop and were transferred to test plates as day 1 adults and imaged after 96 hours. Microscope slides were prepared 1 h prior to microscopy with a 3% agarose mount. The worms were immobilized in 10 μL of 0.5 M sodium azide placed on the agarose pad for 2 min. Pictures were taken immediately after slide preparation using a Leica M165FC dissecting microscope. Worm size comparisons were quantified in ImageJ (Schneider et al., 2012) bundled with 64-bit Java 1.8.0 using polygon tool and saved as macros. Data were plotted by R version 4.1.0, Microsoft Excel 365, and GraphPad Prism.

**Flee rate**

10 gravid adults were placed on new NGM plates and allowed to lay eggs for 4 hours before they were removed. The plates with synchronized eggs were placed back in the 20°C incubator until they reached day 1 of adulthood. 30 worms were transferred to each condition plate and the number of worms that fled was tabulated after 24 hours. A minimum of three plates per condition were used per replicate experiment.

**Pumping rate**

Animals were synchronized by placing 10 N2 gravid adult worms on NGM plates seeded with *E. coli* OP50 and allowing them to lay eggs for 2 hours at 20°C. The gravid adult worms were then removed, and the eggs were allowed to hatch and grow at 20°C until they reached day 1 of adulthood. Worms were transferred to condition plates and the pumping rate was determined after 24 hours. Individual worms were starved for 1 hour, then transferred to food for the assay. The number of contractions of the pharyngeal bulb of 15-20 worms per condition was counted over 30 seconds. A Leica M205C microscope was used with focus on the pharynx.

**Thrashing rate**

Animals were synchronized by placing 10 N2 gravid adult worms on NGM plates seeded with *E. coli* OP50 and allowing them to lay eggs for 2 hours at 20°C. The gravid adult worms were then removed, and the eggs were allowed to hatch and grow at 20°C until they reached day 1 of adulthood. Worms were transferred to condition plates and the thrashing rate was determined after 24 hours. 15-20 worms per condition were transferred to 20 μL of M9 and the thrashing rate of each worm was counted over 30 seconds.

**Lifespan**

Synchronization and preparation of animals for lifespan experiments followed previously published techniques (Sutphin and Kaeberlein, 2009) . Briefly, 15 gravid adults were placed on new NGM plates. After 4 hours the gravid adults were removed and the plates with synchronized eggs were placed back in the 20°C incubator until they reached young adulthood (~2.5 days). Fed conditions- Approximately 60 worms were transferred to fresh 5-Fluoro-2′-deoxyuridine (FUdR) plates seeded with 200 μL live *E. coli* OP50 (OD _600_ 3.0) concentrated 5x on days 3, 4, 5, 7 and 10 from egg. Bacterial Deprivation (BD) conditions- Approximately 60 worms were transferred to fresh fed FUdR plates seeded with 200 μL live *E. coli* OP50 (OD _600_ 3.0) concentrated 5x on days 3 and 4 from egg, then transferred to FUdR plates without food on days 5, 7 and 10 from egg where they remained for the duration of the lifespan. A minimum of two plates per condition were used per replicate experiment. Experimental animals were scored every 2-3 days and considered dead when they did not move in response to gentle prodding with a platinum wire pick under a dissection microscope.

**Statistics and reproducibility**

One-way ANOVA with Tukey Post-Hoc analysis was used to derive p-values for worm size, flee rate, pumping and thrashing comparisons. Log-rank test was used to derive p-value for lifespan comparisons (Han et al., 2024) . All error bars shown in the figures represent the standard error of the mean (SEM).

## References

[R1] Burnett Kyra, Edsinger Eric, Albrecht Dirk R. (2018). Rapid and gentle hydrogel encapsulation of living organisms enables long-term microscopy over multiple hours. Communications Biology.

[R2] Calhoun Adam J, Chalasani Sreekanth H, Sharpee Tatyana O (2014). Maximally informative foraging by Caenorhabditis elegans. eLife.

[R3] Churgin Matthew A, Jung Sang-Kyu, Yu Chih-Chieh, Chen Xiangmei, Raizen David M, Fang-Yen Christopher (2017). Longitudinal imaging of Caenorhabditis elegans in a microfabricated device reveals variation in behavioral decline during aging. eLife.

[R4] Culotti Joseph G, Russell Richard L (1978). OSMOTIC AVOIDANCE DEFECTIVE MUTANTS OF THE NEMATODE
*CAENORHABDITIS ELEGANS*. Genetics.

[R5] Fawcett Emily M., Horsman Joseph W., Miller Dana L. (2012). Creating Defined Gaseous Environments to Study the Effects of Hypoxia on <em>C. elegans</em>. Journal of Visualized Experiments.

[R6] Garigan Delia, Hsu Ao-Lin, Fraser Andrew G, Kamath Ravi S, Ahringer Julie, Kenyon Cynthia (2002). Genetic Analysis of Tissue Aging in
*Caenorhabditis elegans*
: A Role for Heat-Shock Factor and Bacterial Proliferation. Genetics.

[R7] Han Seong Kyu, Kwon Hyunwoo C., Yang Jae-Seong, Kim Sanguk, Lee Seung-Jae V. (2024). OASIS portable: User-friendly offline suite for secure survival analysis. Molecules and Cells.

[R8] Houthoofd K., Johnson T. E., Vanfleteren J. R. (2005). Dietary Restriction in the Nematode Caenorhabditis elegans. The Journals of Gerontology Series A: Biological Sciences and Medical Sciences.

[R9] Kaeberlein Tammi L., Smith Erica D., Tsuchiya Mitsuhiro, Welton K. Linnea, Thomas James H., Fields Stanley, Kennedy Brian K., Kaeberlein Matt (2006). Lifespan extension in
*Caenorhabditis elegans*
by complete removal of food. Aging Cell.

[R10] Kaletsky Rachel, Moore Rebecca S., Vrla Geoffrey D., Parsons Lance R., Gitai Zemer, Murphy Coleen T. (2020). C.&nbsp;elegans interprets bacterial non-coding RNAs to learn pathogenic avoidance. Nature.

[R11] Mohammadi Aylia, Byrne Rodgers Jarlath, Kotera Ippei, Ryu William S (2013). Behavioral response of Caenorhabditis elegansto localized thermal stimuli. BMC Neuroscience.

[R12] Moyson Sofie, Town Raewyn M., Vissenberg Kris, Blust Ronny (2019). The effect of metal mixture composition on toxicity to C. elegans at individual and population levels. PLOS ONE.

[R13] Moyson Sofie, Vissenberg Kris, Fransen Erik, Blust Ronny, Husson Steven J. (2017). Mixture effects of copper, cadmium, and zinc on mortality and behavior of
*Caenorhabditis elegans*. Environmental Toxicology and Chemistry.

[R14] Oh Seung-Il, Park Jin-Kook, Park Sang-Kyu (2015). Lifespan extension and increased resistance to environmental stressors by N-Acetyl-L-Cysteine in Caenorhabditis elegans. Clinics.

[R15] Olsen Anders, Vantipalli Maithili C., Lithgow Gordon J. (2006). Lifespan extension of Caenorhabditis elegans following repeated mild hormetic heat treatments. Biogerontology.

[R16] OLSEN ANDERS, VANTIPALLI MAITHILI C., LITHGOW GORDON J. (2006). Using
*Caenorhabditis elegans*
as a Model for Aging and Age‐Related Diseases. Annals of the New York Academy of Sciences.

[R17] Pittman William E., Sinha Drew B., Zhang William B., Kinser Holly E., Pincus Zachary (2017). A simple culture system for long-term imaging of individual C. elegans. Lab on a Chip.

[R18] Schneider Caroline A, Rasband Wayne S, Eliceiri Kevin W (2012). NIH Image to ImageJ: 25 years of image analysis. Nature Methods.

[R19] Smith Erica D, Kaeberlein Tammi L, Lydum Brynn T, Sager Jennifer, Welton K Linnea, Kennedy Brian K, Kaeberlein Matt (2008). Age- and calorie-independent life span extension from dietary restriction by bacterial deprivation in Caenorhabditis elegans. BMC Developmental Biology.

[R20] Song Shaojuan, Guo Yaping, Zhang Xiaomin, Zhang Xueyao, Zhang Jianzhen, Ma Enbo (2014). Changes to Cuticle Surface Ultrastructure and Some Biological Functions in the Nematode Caenorhabditis Elegans Exposed to Excessive Copper. Archives of Environmental Contamination and Toxicology.

[R21] Stiernagle Theresa (2006). Maintenance of C. elegans. WormBook.

[R22] Sutphin George L., Kaeberlein Matt (2009). Measuring <em>Caenorhabditis elegans</em> Life Span on Solid Media. Journal of Visualized Experiments.

[R23] Tissenbaum Heidi A. (2014). Using
*C. elegans*
for aging research. Invertebrate Reproduction & Development.

[R24] Yuen Grace J., Ausubel Frederick M. (2018). Both live and dead
*Enterococci*
activate
*Caenorhabditis elegans*
host defense via immune and stress pathways. Virulence.

[R25] Zhao Lina, Fenk Lorenz A., Nilsson Lars, Amin-Wetzel Niko Paresh, Ramirez-Suarez Nelson Javier, de Bono Mario, Chen Changchun (2022). ROS and cGMP signaling modulate persistent escape from hypoxia in Caenorhabditis elegans. PLOS Biology.

